# Asian and African lineage Zika viruses show differential replication and innate immune responses in human dendritic cells and macrophages

**DOI:** 10.1038/s41598-019-52307-1

**Published:** 2019-10-31

**Authors:** Pamela Österlund, Miao Jiang, Veera Westenius, Suvi Kuivanen, Riia Järvi, Laura Kakkola, Rickard Lundberg, Krister Melén, Miša Korva, Tatjana Avšič – Županc, Olli Vapalahti, Ilkka Julkunen

**Affiliations:** 1 Expert Microbiology Unit, Finnish Institute for Health and Welfare, 00300 Helsinki, Finland; 2 Department of Virology, University of Helsinki, 00290 Helsinki, Finland; 3 Institute of Biomedicine, University of Turku and Turku University Hospital, 20520 Turku, Finland; 4 Faculty of Medicine, Institute of Microbiology and Immunology, University of Ljubljana, 1000 Ljubljana, Slovenia

**Keywords:** Cytokines, Innate immune cells, Virus-host interactions, Infection

## Abstract

Zika virus (ZIKV) infections in humans are considered to be mild or subclinical. However, during the recent epidemics in the Pacific Islands and the Americas, the infection was associated with Quillain-Barré syndrome and congenital infections with fetal brain abnormalities, including microcephaly. Thus, more detailed understanding of ZIKV-host cell interactions and regulation of innate immune responses by strains of differential evolutionary origin is required. Here, we characterized the infection and immune responses triggered by two epidemic Asian/American lineage viruses, including an isolate from fetal brains, and a historical, low passage 1947 African lineage virus in human monocyte-derived dendritic cells (DCs) and macrophages. The epidemic Asian/American ZIKV replicated well and induced relatively good antiviral responses in human DCs whereas the African strain replicated less efficiently and induced weaker immune responses. In macrophages both the African and Asian strains showed limited replication and relatively weak cytokine gene expression. Interestingly, in macrophages we observed host protein degradation, especially IRF3 and STAT2, at early phases of infection with both lineage viruses, suggesting an early proteasomal activation in phagocytic cells. Our data indicates that ZIKV evolution has led to significant phenotypic differences in the replication characteristics leading to differential regulation of host innate immune responses.

## Introduction

Zika virus (ZIKV) belongs to the family of *Flaviviridae*, which is a large group of viruses including e.g. Dengue, West Nile, Japanese encephalitis, Tick-borne encephalitis and many other important human pathogenic viruses. In most individuals ZIKV infection is a self-limiting disease with fever, rash, arthritis, conjunctivitis, malaise and headache with perhaps 80% of infected individuals suffering from a very mild or a subclinical infection^1^. However, in 2015 there was increasing evidence that ZIKV is linked to congenital infections leading to spontaneous abortions and severe neonatal birth defects^2,3^. Indeed, it was observed that maternal ZIKV infection can spread to the fetal brains causing the death of developing neurons, brain calcification and cortical displacement leading to reduced brain volume and development of microcephaly^4,5^. In addition, ZIKV infections have been associated with Guillain-Barré syndrome and likely other milder neurological symptoms that may appear in early childhood^6^. Due to the rapid spread of ZIKV infections in the Americas and accumulating data on severe birth defects in children whose mothers had suffered a ZIKV infection during pregnancy, WHO declared ZIKV infection as a global health alert during the epidemic in 2015–2016^7^.

ZIKV was identified in *Rhesus* monkeys in Uganda and the virus was isolated in 1947^8^. Later on, it became evident that many *Aedes* (Stegomyia) species mosquitoes are transmitting the virus to primates and humans^9^. Initially, the ZIKV appearance was restricted to certain areas in Africa and later on in Asia, but in recent years the virus has spread widely in the tropical and subtropical areas in the world. The virus follows well the geographic distribution of *Aedes* species mosquitoes such as *A*. *africanus*, *A*. *albopictus* and *A*. *aegyptii*, which are the vectors for other important flaviviruses as well. During the last ten years, ZIKV spread to Southeast Asia and the Polynesian Islands followed by an extremely rapid spread of the virus in Southern and Central America in 2015–2016, including the southern parts of the United States^1,10–12^. During its global spread, the virus has undergone marked genetic evolution and presently ZIKV strains have been classified as East African, West African and Asian lineages, the latter of which includes the virus strains isolated in the Americas^13^. At present, there is little information whether the viruses from different lineages show significant differences in pathogenesis or in their ability to replicate in human cells.

Innate immunity plays an important role in the early phases of microbial infections including viral infections. The key cell types regulating innate immunity include dendritic cells (DCs) and macrophages. These cells reside in tissues where they are on alert for invading pathogens. DCs take up microbes, microbial components or they are infected by viruses, which leads to the activation of the cells, increased cytokine production and migration of activated DCs into the local lymph nodes where they present microbial antigens to T cells^14^. DCs thus operate as an important cell type regulating both innate and adaptive immunity. Macrophages, instead, reside in tissues where they act mainly locally by taking up and destroying microbes as well as by secreting inflammatory cytokines and other inflammatory mediators^15^. Although immature DCs in humans have been shown to be permissive to ZIKV infection, there is still some controversy on the impact of lineage differences on the cell-type specific infectivity and regulation of innate immunity^16–18^. Macrophages, which are readily infected by many flaviviruses like Dengue and West Nile viruses^19,20^, have been mostly neglected as a potential target for ZIKV infection.

In the present study, we have characterized Zika virus infection in primary human monocyte-derived DCs and macrophages. Firstly, we evaluated the susceptibility of human immune cells to a ZIKV infection. Secondly, we compared the infection pattern of Asian lineage fetal brain and blood ZIKV isolates and a historical African strain (initially a monkey isolate) in both cell types. We observed that the recent Asian/American lineage Zika viruses replicated well in DCs but poorly in macrophages, while the African lineage virus showed limited infectivity in both cell types. The ability of the virus to replicate correlated well with the magnitude of innate antiviral gene expression and cytokine production. However, all ZIKV strains analyzed induced innate immune responses in such a level that an antiviral state, as evidenced by enhanced expression of antiviral MxA protein, was established in both studied human cell types.

## Results

### Infection of human DCs and macrophages by Zika viruses of different evolutionary lineages

**Figure 1:**
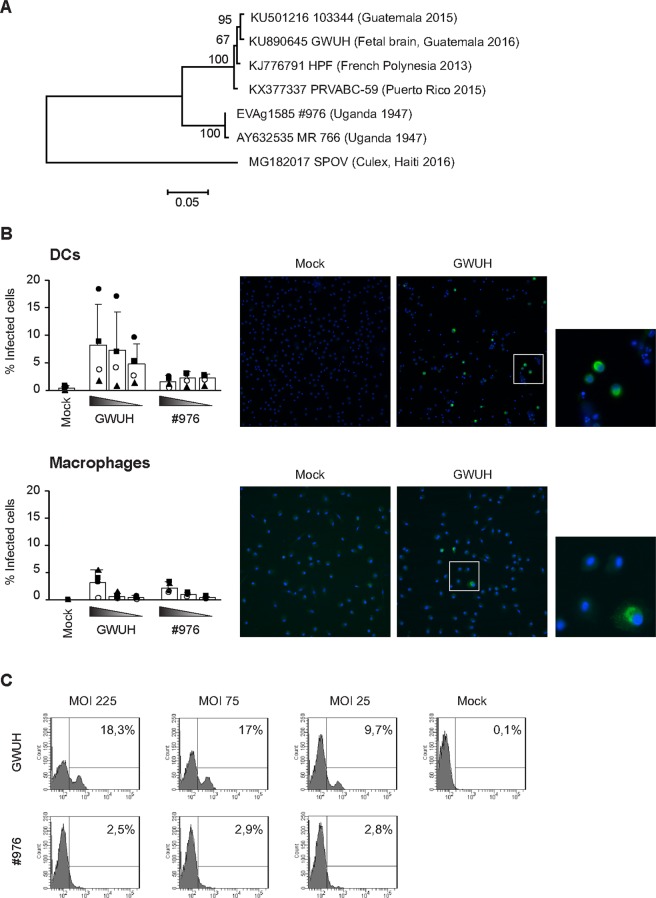
Susceptibility of human monocyte-derived dendritic cells (DCs) and macrophages to fetal brain-isolated Asian lineage ZIKV and historical African lineage ZIKV infection. (**A**)  Phylogenetic analysis of complete coding regions of the Asian and African ZIKV strains used in this study. Maximum Likelihood tree based on Tamura-Nei model with bootstrap support of 1000 replicates constructed using MEGA7 is drawn to scale with branch lengths measured in the number of substitutions per site. The percentage of replicate trees clustered together is shown next to the branches. Viral strains are shown with their name and place of origin. (**B**) Flow cytometric and fluorescence microscopic analysis of ZIKV-infected DCs or macrophages. Cells were infected with Asian lineage GWUH or African lineage #976 strains at high MOI values of 25, 75 or 225 TCID_50_/cell and the cells were fixed at 48 h after infection. Data is presented as mean +/− SEM of cells generated from 4 independent blood donors. Individual donors are shown with different symbols. Immunofluorescence images of representative mock and GWUH-infected cells at a MOI of 225 TCID_50_/cell. Blue: DAPI, green:anti-ZIKV NS3 antibody staining. (**C**) Representative histogram patterns from flow cytometric analysis of GWUH or #976 virus-infected DCs from one donor.

In this study we analysed the infection profiles of ZIKV strains of African and Asian lineages in human innate immune cell models. A recent Asian lineage ZIKV strain (infection contracted in Guatemala) isolated from fetal brains (ZIKV-FB-GWUH-2016^5^), another Asian strain isolated from a viremic adult patient who contracted the infection in French Polynesia (ZIKV-H-PF-2013) and an ancestor, low-passage African strain (ZIKV-#976-Uganda-1947) were used. As shown in Fig. 1A, GWUH is an Asian lineage virus and genetically very closely related to the 2015 epidemic Guatemala strains^5^. The HPF strain is very closely related to the 2015–2016 epidemic strains of the Americas of which an isolate from Puerto Rico in 2015 (PRVABC59) is widely used as a reference strain for the epidemic^13^. The genetic difference between the recent epidemic strains and the original African lineage viruses is ca. 6% (Fig. 1A).

In order to define the susceptibility of human immune cells to ZIKV infection, we infected primary human monocyte-derived DCs and macrophages with the GWUH strain at MOI of 5 (TCID_50_/cell) and evaluated the frequency of virus-infected cells at 48 h post infection (p.i.). In this time point we saw hardly any virus-positive DCs by flow cytometric analysis or macrophage infection by immunofluorescent microscopy when an in-house produced anti-ZIKV NS3 specific antibody was used. The specificity and sensitivity of anti-ZIKV NS3-specific antibodies was excellent in GWUH-infected Vero E6 cells and no background staining was seen in immunofluorescence analysis (Supplementary Fig. S1). To further evaluate ZIKV infection in DCs and macrophages we infected the cells with high doses of Asian lineage GWUH and African lineage #976 strains and analysed the percentage of virus-infected cells by immunofluorescence staining at 48 h p.i. In DCs very high doses of GWUH ZIKV(MOI 25-225) lead to a clearly detectable infection in 2–18% of the cells in different donors (Fig. 1B,C). However, in African lineage ZIKV #976 virus-infected DCs, only a few percentage of cells showed evidence of virus protein expression (Fig. 1B,C). In macrophages the infectivity rate remained low with both virus strains (Fig. 1B). The data indicates that DCs are more permissive to the Asian ZIKV strains, whereas macrophages show low susceptibility to both Asian and African ZIKV strains.

### Replication of ZIKV in human DCs and macrophages

**Figure 2:**
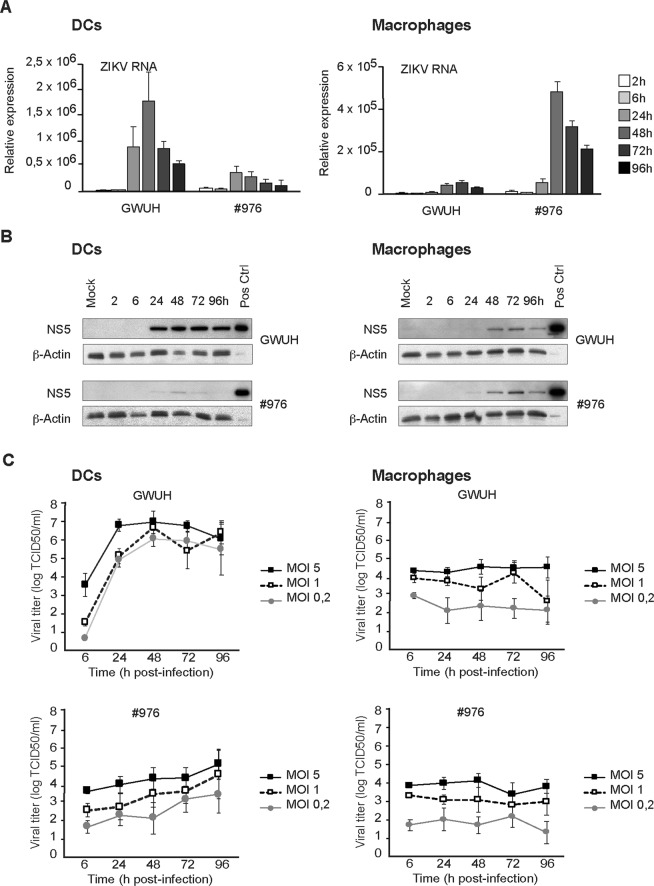
Comparison of virus replication in human DCs and macrophages infected with Asian and African lineage ZIKV strains. The Asian lineage GWUH strain or the African lineage #976 ZIKV strain was used to separately infect human DCs or macrophages from four different donors. (**A**,**B**) Cells were infected by the viruses at MOI of 75 TCID_50_/cell and collected at different times after infection as indicated in the figure. (**A**) ZIKV RNA expression was quantitated by qRT-PCR using NS5 gene-specific probes. Data is presented as mean +/− SEM from four individual donors and a representative experiment out of two is shown. (**B**) ZIKV-infected cells from the same 4 donors were collected at different times after infection, cells were pooled and proteins were separated on SDS-PAGE, immunoblotted and stained with anti-NS5 and anti-β-Actin antibodies. ZIKV-infected Vero E6 cell extract was used as a positive control in immunoblotting. (**C**) For the measurement of infectious ZIKV production into cell supernatants, cells were infected with low doses (MOI values 0.2, 1 or 5 TCID_50_/cell) of GWUH or #976 strains followed by collection of cell culture supernatants at times indicated in the figure. Viral titers were measured from the supernatant samples using the end-point dilution assay in Vero E6 cells (TCID_50_/ml) and the virus titers were calculated using the Spearman-Karber method. Results represent the means +/− SEM from DC or macrophage cultures from four different blood donors.

As human DCs and macrophages were found to be rather weakly permissive to ZIKV infection even with an extremely high doses of viruses, we systematically evaluated the replication efficiency of the Asian lineage fetal brain isolate GWUH strain and the low passage African lineage strain #976 in these cell types. At high MOI values of 75 TCID_50_/cell, viral RNA and protein expression readily increased with peak values seen at 24–48 h p.i. in DCs and at 48–72 h p.i. in macrophages (Fig. 2A,B). The Asian lineage GWUH Zika virus replicated better in DCs compared to that in macrophages, and strong viral protein expression was seen in infected DCs. Western blot analysis showed that the #976 virus replicated to some extent both in DCs and macrophages (Fig. 2B). Altogether, by comparing the ZIKV strains from African and Asian lineages, the data indicates that different ZIKV strains show clear differences in their replication and viral RNA and protein expression patterns in primary human leukocytes.

In order to evaluate the capability of ZIKV strains to replicate and produce new progeny viruses, we challenged the cells with low doses of ZIKV GWUH and #976 viruses (MOI 0.2, 1 or 5 TCID_50_/cell). To analyze the productivity of the infection, cell culture supernatants were collected at different time points after infection and viral titers were determined in Vero E6 cells with an end-point dilution assay. Already at 24 h after the infection, GWUH virus-infected DCs were found to produce high levels of infectious viruses and the viral titers ranged from 10^4^ to almost 10^7^/ml depending on the input MOI values (Fig. 2C). In #976 virus-infected DCs the virus production was slower and the amount of secreted viruses continued to increase up to 96 h after infection reaching TCID_50_ values of 10^3^ to 10^5^/ml (Fig. 2C). In macrophages, instead, the productivity of ZIKV infection was less efficient and secreted virus titers remained at low, similar to inoculum virus levels ranging from 10^2^ to 10^4^ TCID_50_ values/ml (Fig. 2C). Even though there were differences in the replication of ZIKV in cells obtained from different blood donors, ZIKV was able to replicate both in DCs and macrophages. However, clearly detectable increases in ZIKV production was only seen in DCs (Fig. 2).

### Activation of innate cytokine responses in ZIKV-infected human DCs and macrophages

**Figure 3:**
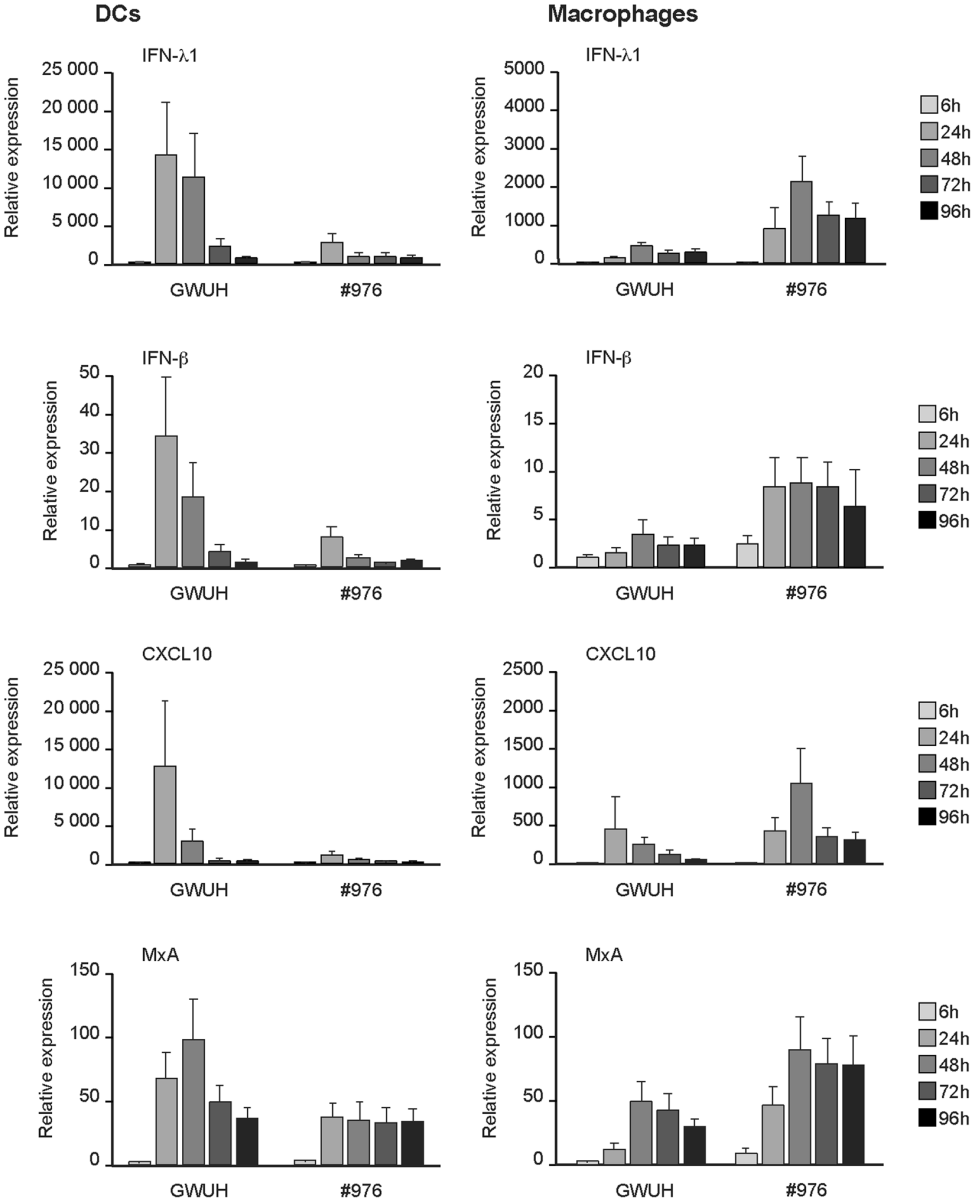
Differential induction of cytokine mRNA expression in human DCs and macrophages infected with Asian and African lineage ZIKV strains. Human monocyte-derived DCs or macrophages obtained from four different blood donors were separately infected with a fetal brain-isolated GWUH strain (Asian lineage) or with an African lineage #976 ZIKV strain at MOI of 75 TCID_50_/cell. The cells were collected at different times after infection and total cellular RNA was isolated. The expression of IFN-λ1, IFN-β, CXCL10 and MxA mRNA was analyzed by qRT-PCR. The mRNA levels are presented as fold induction compared to the mock sample. The data (means +/− SEM) shown is a representative experiment out of two independent experiments.

Since the Asian lineage ZIKV GWUH appeared to grow well in DCs but inefficiently in macrophages, and the African lineage ZIKV #976 showed weak replication in both cell types, we addressed the question whether these viruses would also show differences in innate immune responses they induced. With a high MOI infection of 75 TCID_50_/ml both viruses induced IFN-λ1, IFN-β and CXCL10 mRNA expression relatively well in DCs with GWUH strain showing ca. 10-fold higher cytokine mRNA levels than the African #976 strain (Fig. 3). In macrophages, instead, the infection with the African ZIKV #976 strain led to somewhat higher IFN-λ1, IFN-β and CXCL10 mRNA expression levels as compared to those induced by the GWUH strain. It is of interest that the fold induction of cytokine mRNAs by the #976 strain was at similar levels in DCs and macrophages, while there was a dramatic difference in GWUH virus induced responses between DCs and macrophages in favor of DCs (Fig. 3). Altogether, cytokine gene expression levels seemed to follow viral RNA expression patterns (compare Figs 2 and 3). Interestingly, type I and type III IFN-inducible MxA gene expression did not show very dramatic differencies between the viruses and cell types, which is likely explained by the fact that in primary human leukocytes MxA gene is induced with very low doses of IFNs^21,22^.

**Figure 4:**
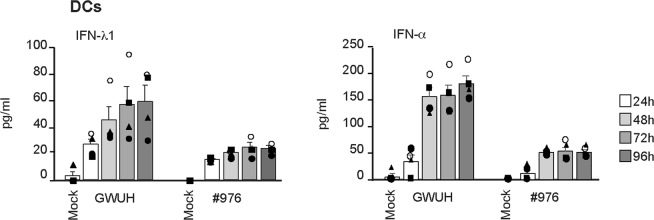
Production of IFNs from ZIKV-infected DCs. For the measurement of secreted IFN-λ1 and IFN-α protein levels with enzyme immunoassay (EIA), cell culture supernatants were collected at different times after infection with GWUH or #976 strains at MOI of 75 TCID_50_/cell. The samples were analysed in duplicates in EIA assay. The supernatants from ZIKV-infected DCs (obtained from 4 donors) were analysed separately and the results are presented as the mean values +/− SEM and the data of individual donors are shown with different symbols.

We also determined the amounts of secreted IFN-λ1 and IFN-α from virus-infected cells by cytokine-specific EIAs. GWUH induced clearly higher levels of secreted type I and type III IFNs in DCs as compared to those seen by the #976 virus (Fig. 4). ZIKV-infected macrophages produced extremely low levels of IFNs into cell culture supernatants and they were found only in late time points of infection (data not shown). Altogether, the secreted levels of IFNs were in good agreement with Zika virus replication and virus-induced cytokine mRNA levels in different types of cells (Figs 2–4).

### Activation of IRF3 and STAT2 signaling molecules in ZIKV-infected human DCs and macrophages

**Figure 5:**
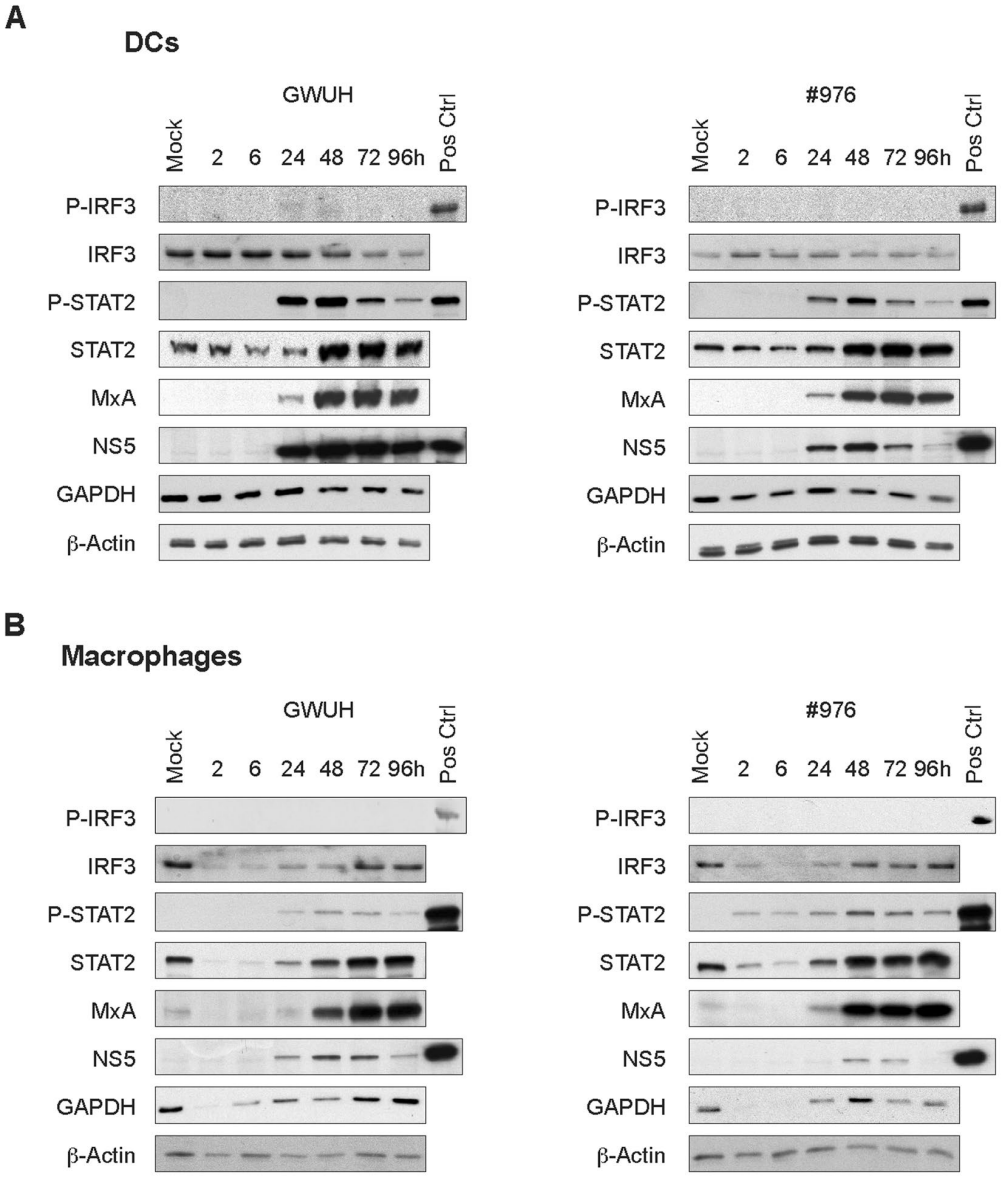
Expression of signaling molecules and antiviral proteins in ZIKV-infected human DCs and macrophages. Human primary (**A**) DCs and (**B**) macrophages were infected with GWUH or #976 ZIKV strains at a MOI of 75 TCID_50_/cell and the cells from four individual blood donors were collected at different time points after infection, pooled and cellular protein samples were prepared for immunoblotting. Uninfected mock samples were used as negative control and influenza A virus-infected cells (MOI 1, 24 h infection) as positive control samples. The expression levels of phospho-IRF3 (P-IRF3), IRF3, phospho-STAT2 (P-STAT2), STAT2 and antiviral MxA proteins were analyzed by immunoblotting using specific antibodies. The expression of ZIKV NS5 protein was analyzed for visualizing the virus replication with ZIKV-infected DCs and macrophages. ZIKV infected (GWUH) Vero E6 cell lysate functioned as a positive control, and GAPDH and β-Actin were stained as loading controls. The data shown is a representative experiment out of two independent experiments (altogether n = 8).

Since both the Asian and African lineage Zika viruses induced cytokine gene expression in human DCs and also weakly in macrophages, we analyzed the level of phosphorylation of IRF3 and STAT2 in response to ZIKV infection. In addition, the expression of IRF3, STAT2 and antiviral MxA proteins were also analysed. In Asian lineage GWUH virus-infected DCs, we could only detect a very weak signal corresponding to the phosphorylated form of IRF3 (P-IRF3), while in influenza A virus-infected cells (a positive control) P-IRF3 was readily detectable (Fig. 5A). Starting at 24 h after infection, we observed the appearance of phospho-STAT2 (P-STAT2) and an enhanced expression of STAT2, MxA and ZIKV NS5 proteins. In DCs the African #976 ZIKV strain was also able to induce the phosphorylation of STAT2 (P-STAT2) but no P-IRF3 was detectable. The expression of STAT2, MxA and ZIKV NS5 protein was enhanced starting at 24 h after infection with the #976 virus. As a whole, the observation is indicative of efficient type I and type III IFN secretion (IFN-α/β and IFN-λs, respectively), which are known to activate and phosphorylate STAT2 and enhance the expression of STAT2 and MxA, which are well-known IFN-inducible genes^21,23^.

In macrophages, we could not find any detectable P-IRF3 in GWUH or #976 ZIKV-infected cells. Interestingly, in macrophages, the expression of total IRF3 or STAT2 protein levels seemed to be reduced at 2 and 6 h after infection with both types of viruses (Fig. 5B). However, at later times of infection (24–48 h) the expression of IRF3 returned back to basal levels and the expression of STAT2 was clearly enhanced. An early IRF3 and STAT2 degradation was induced by both the African #976 and the Asian GWUH strains irrespective of their differential growth or cytokine responsiveness in macrophages (Figs 1–4). In addition, STAT2 was phosphorylated and the expression of both STAT2 and MxA proteins was seen starting at 24 h after the infection with either one of the viruses indicating that at least some IFN production took place in macrophages in response to ZIKV infection (Fig. 5B). It was of interest that some P-STAT2 was detectable in macrophages at 2–6 h after infection with the #976 virus (Fig. 5B right panel). This could be due to residual amounts of IFNs in Vero E6 cell-produced ZIKV stocks, albeit Vero cells are considered to be IFN-deficient^24^. Indeed, some IFN-λ1 (<100 pg/ml) was detectable by IFN-specific EIA in ZIKV stocks. In most experiments the stocks were diluted to levels where STAT2 phosphorylation did not take place (Fig. 5).

### Characterization of ZIKV infection and activation of innate immunity induced by Asian ZIKV strains of different origin

**Figure 6:**
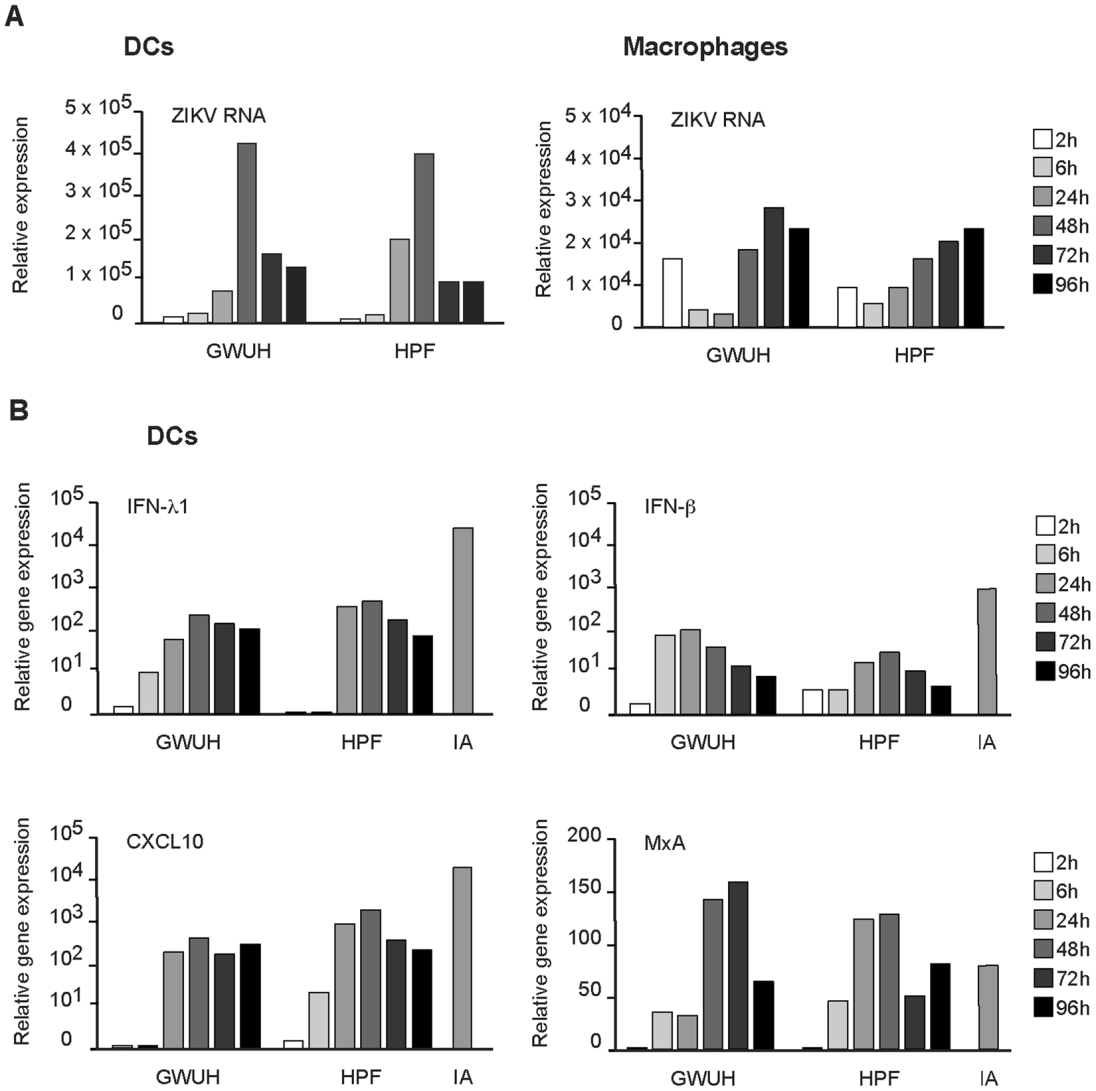
Expression of ZIKV RNA and host innate immune genes in human DCs and macrophages infected with two recent Asian lineage ZIKV strains. Differentiated DCs and macrophages from four independent blood donors were separately infected with GWUH or HPF strains at MOI of 2 and analyzed for the expression of viral RNA (panel A) or host cell antiviral genes (panel B) at different times after infection during a 4-day infection experiment. (**A**) For viral RNA expression cells were collected at different times after infection, cells from different donors were pooled and total cellular RNA was extracted and qRT-PCR analysis for ZIKV RNA (using NS5 gene as a target) was carried out. (**B**) The DCs from different donors were collected, pooled and total cellular RNA was isolated followed by quantitation of IFN-λ1, IFN-β, CXCL10 and MxA mRNA expression by qRT-PCR. As a positive control cells were infected with influenza A/Beijing/353/89 virus at MOI of 1 for 24 h. The results are shown as fold induction over the mock sample and the data is representative of two individual experiments (altogether n = 8).

Next we compared the ability of two recent Asian lineage ZIKV isolates, the fetal brain isolate GWUH and HPF, a virus isolated from the serum specimen of an adult patient (ZIKV-H-PF-2013), to replicate and induce cytokine responses in human DCs and macrophages. Cells were infected at MOI of 2 for different time periods followed by isolation of total cellular RNA and qRT-PCR analysis to quantitate ZIKV RNA expression. Due to a considerable variability between the cells of different blood donors (Fig. 1B)^17^, virus-infected cells of different donors were pooled for RNA analysis in order to get a more global and general view of the infection kinetics. In DCs, both Asian Zika virus strains appeared to replicate with a similar efficacy (Fig. 6A). In ZIKV-infected macrophages, the expression of viral RNA was increased to some extent, but the levels remained much lower (one log) compared to viral RNA levels seen in DCs (Fig. 6A). The data clearly shows that, albeit both ZIKV strains are able to infect both human DCs and macrophages, virus replication is better in DCs.

Since the two analysed Asian lineage ZIKV strains appeared to replicate equally well in DCs and macrophages we analysed whether they also induced cytokine gene expression equally well. In DCs, both viruses were able to induce mRNA expression of IFN-λ1, IFN-β, CXCL10 and IFN-inducible MxA genes apparently at similar rates (Fig. 6B). Induced cytokine mRNAs were seen already 6 h after infection and the induced levels peaked at 24–48 h p.i. Quantitative RT-PCR analyses revealed induced cytokine and MxA mRNA levels to range from 100 to 1000-fold over the basal levels. However, in DCs ZIKV-induced cytokine mRNA levels remained approximately 10 to 100-fold lower levels compared with those induced by influenza A virus (Fig. 6B). It has to be pointed out that in general, influenza A virus is an excellent inducer of cytokine gene expression in human primary DCs and macrophages^21,25^. In macrophages, the expression of cytokine genes remained close to the undetectable levels with both Asian lineage viruses (data not shown).

### ZIKV is highly sensitive to the antiviral actions of type I IFNs

**Figure 7:**
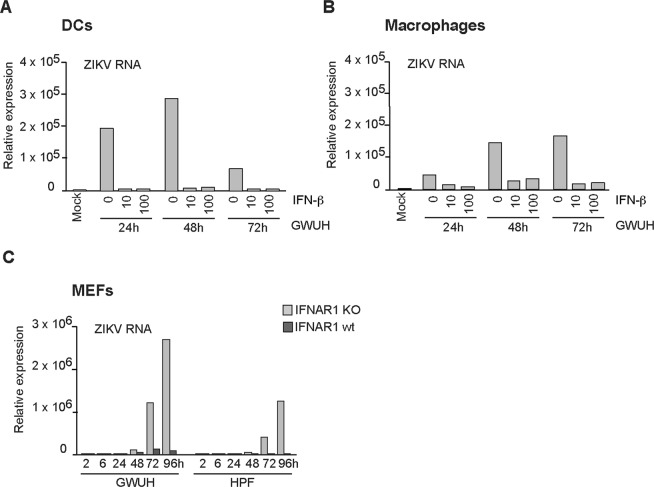
ZIKV is highly sensitive to the antiviral activity of type I IFNs. Monocyte-derived human (**A**) DCs or (**B**) macrophages from 4 individual blood donors were primed with different doses of IFN-β (0, 10 or 100 IU/ml) for 16 h followed by infection with the ZIKV GWUH strain (MOI 2). Cells were collected at different time points after infection and samples from different donors were pooled for isolation of total cellular RNA. Virus replication was analyzed by quantitating viral RNA by qRT-PCR. The results are presented as relative expression over the mock sample without cytokine stimulation or virus infection. (**C**) Mouse embryonal fibroblasts (MEFs) from wild-type mouse (IFNAR1 wt) and from IFN-α receptor chain 1 knock-out (*Ifnar-1*−/−; IFNAR1 KO) mouse were infected with GWUH or HPF strains of ZIKV at MOI of 2 and cells were harvested at different time points after infection followed by isolation of total cellular RNA. Viral RNA expression was quantitated by qRT-PCR, and results are shown as relative expression over the uninfected sample.

Next, we addressed the question whether the recent Asian lineage Zika viruses are sensitive to the antiviral effects of type I IFNs (IFN-β). Human DCs or macrophages were pretreated with recombinant human IFN-β with 10 or 100 IU/ml followed by infection with GWUH ZIKV (MOI 2). At 24, 48 and 72 h after the infection cells were collected, total cellular RNA was isolated and ZIKV RNA expression was analyzed by qRT-PCR. Pretreatment with as low as 10 IU/ml of IFN-β led to almost complete inhibition of ZIKV replication in DCs (Fig. 7A). In macrophages, ZIKV replication was also dramatically reduced by IFN-β pretreatment even though some residual viral RNA expression remained (Fig. 7B). This suggests that, in accordance with previous studies done in human DCs, human skin fibroblasts and human lung epithelial cell line A549^17,18,26^, in our primary immune cell models the epidemic ZIKV was highly sensitive to the antiviral actions of type I IFNs.

Since it was apparent that type I IFNs played an important role in inhibiting the replication of ZIKV, we conducted *in vitro* infection experiments in wild-type or type I IFN receptor chain 1 (IFNAR1) knock-out mouse embryonal fibroblasts (MEFs). MEFs were productively infected by the Asian lineage GWUH or HPF Zika viruses and the expression of ZIKV RNA, as analyzed by qRT-PCR, was very high in IFNAR1 KO cells as compared to the wild-type MEFs (Fig. 7C). Viral RNA levels were approximately 50–100-fold higher in IFNAR1 KO cells compared to those seen in wild-type cells, indicating an important role of type I IFNs in restricting the infection in cell culture.

## Discussion

The ZIKV epidemic in the Americas and its association with congenital defects like microcephaly raised a global infection alert. The characterization of ZIKV infection and the immune regulation induced by the infection have been studied in different *in vitro* cell lines as well as in type I IFN receptor knockout mouse model^27^. However, the studies in primary human immune cell models have remained rare. In the present study, we have demonstrated that virus strains from different ZIKV lineages show differential replication capacity and ability to induce innate immune responses in human monocyte-derived DCs and macrophages. We observed a productive infection in DCs with a recent epidemic ZIKV strain, while virus replication remained at a very low level in human macrophages as noted by low viral RNA and protein expression. Despite that, a clear antiviral state was likely established in response to virus infection as we observed marked MxA expression in macrophages infected with the Asian Zika virus strain. However, both cell types were as permissive to the African lineage virus and virus replication led to the activation of innate immune responses. Thus, we observed clear differences in virus strains of differential evolutionary origin in their ability to replicate and induce innate immune responses in primary human immune cells.

The reports of human infections with ZIKV remained sporadic until the outbreak in Yap Island in 2007 which proceeded with a rapid virus spread through the Pacific Islands to Southern and Central Americas in 2013–2015^1,13^. The absence of monkeys in the French Polynesian islands suggests that humans must have served as the amplification host for ZIKV during that epidemic^28^. The possibility that birds could transfer the virus along their migration routes for long distances is still unclear^29^. Also, the neurotropic clinical picture of ZIKV infection suggests that the increased pathogenicity may, at least partly, be due to evolutionary changes in the virus. In the present study, our goal was to compare the infection of a historical, low passage African strain and two recent epidemic strains, the other one of which was isolated from fetal brains. Previously, it has been shown that the fetal brain isolate GWUH has a replicative advantage over other related epidemic strains or the prototype African strain MR766 in some cell lines, such as in glioma cells or in microvascular endothelial cells of the newborn^30^. The GWUH strain has 10 amino acid differences from the HPF strain^30^, the other Asian strain used in the present study. However, these changes apparently did not affect the growth properties of the viruses in immune cells since both viruses showed similar growth kinetics and comparable innate immune responses (Fig. 6). This suggests that although the GWUH virus strain was isolated from the brains and it was likely adapted to fetal neuronal cells, it still maintains the ability to replicate in adult peripheral blood cells. The prototype African strain MR766 has been passaged several times in various laboratory models (suckling mouse brains and cell lines) and thus the virus shows adaptation to cell culture environment^31^. Therefore, we used another ancestral African lineage isolate, #976 which was available as a low passage mouse brain isolate (3 passages) followed by cultivation in Vero E6 cells (4 passages). This virus, which was initially a monkey isolate, showed replication in both DCs and macrophages in contrast to the recent epidemic Asian strains which showed attenuated replication in human macrophages (Figs 1–2). In line with this, the only available studies on human blood-derived macrophages show that this cell model is permissive to a recent ZIKV strain (PRVABC59)^32,33^. Moreover, Khaibouillina and co-workers compared the infectivity of African and Asian strains and found, in accordance with us, a higher susceptibility of macrophages to the African ZIKV strain^32^. In DCs, Bowen and co-workers observed a faster infection kinetics with the African strain compared to that of the Asian lineage virus^17^. However, Vielle and co-workers did not find lineage-specific differences in infection profiles^16^. In our study, GWUH and #976 both induced IFN gene expression and cytokine secretion correlated with the magnitude of virus replication, but antiviral MxA expression was observed at similar levels (Figs 3–5). Similar findings were reported by Bowen and co-workers although they showed reduced IFN secretion suggesting that ZIKV could block the translation of IFN mRNAs but yet induce an antiviral state^17^. This observation can, however, be explained by a possibility that small amounts of secreted type I and type III IFNs were rapidly used up by the cells to induce an antiviral response. Our data rather points to that direction since ZIKV induced IFN gene expression at a much lower level as compared to our positive control virus, influenza A virus, but yet the expression of antiviral MxA protein was readily induced by both viruses. It is of note that in the study of Bowen and co-workers a cell culture-adapted MR766 strain was used and thus the comparison to low passage epidemic strains may be difficult. Collectively, the published and our data indicate that ZIKV is able to induce IFN and IFN-induced antiviral gene expression in human immune cells, but the recent Asian-American lineage ZIKV strains have evolved to show restricted replication in human macrophages.

Given that the maternal ZIKV infection can be transmitted vertically to the developing fetus^4,5^, several studies have thus focused on identifying the target cells of ZIKV infection in the placenta and the uterus. Several placental cell types have been shown to be permissive to ZIKV infection, including placental fibroblast, human umbilical vein endothelial cells (HUVEC), placental trophoblasts and placental macrophages (Höfbauer cells)^34^. Interestingly, human placental macrophages showed a more productive infection with recent epidemic ZIKV strains compared with the placental trophoblasts^35^, which have been postulated to transmit resistance to ZIKV infection both in an autocrine and paracrine manner by secreting antiviral IFN-λ1^36^. There is evidence for an important role of IFN-λs in epithelial/endothelial-immune cell barrier against several human viral pathogens, including Dengue virus^37^, influenza A virus^38^, as well as other viruses infecting the respiratory^39^ or gastrointestinal tract^40^. In accordance with previous studies on macrophages^32,33^, we also found out that human blood monocyte-derived macrophages can be infected with recent ZIKV isolates, even though the level of virus replication and host IFN gene expression remains at a very low level. Quicke and colleagues showed that placental macrophages were highly permissive to the epidemic ZIKV strains which induced the production of IFNs^35^. In addition, the study by Foo and co-workers shows that during pregnancy, the primary target cells for ZIKV infection in *in vitro* whole blood cell infection are CD14+ monocytes^41^. Furthermore, the same authors demonstrated that an Asian lineage ZIKV infection promoted the differentiation of non-classical M2-type, IL-10-expressing immunosuppressive monocytes by inhibiting the IFN pathway. However, the infection with an African ZIKV strain led to monocyte differentiation to M1-type inflammatory responses with increased IFN-induced CXCL10 production^41^. This is well in line with our macrophage results, where the early African lineage virus was able to induce innate immune responses (IFN-λ1, IFN-β and CXCL10 expression) but innate immunity was more weakly induced by the Asian ZIKV strain (Fig. 3). These findings may provide insights into immunological differences between early African and recent Asian-American ZIKV strains which may be associated with increased frequency of congenital infections.

There is evidence that the primary target cells of ZIKV infection, skin fibroblasts and DCs are highly permissive to ZIKV, but yet the infection induces only weak immune responses^16–18^. This suggests that ZIKV must have efficient ways to antagonize virus-induced IFN signaling. Recent studies from many groups have postulated different molecular mechanisms by which ZIKV evades host’s IFN-mediated antiviral protection during the infection. The most convincing evidence is that ZIKV NS5 is interfering with IFN signaling by inducing the degradation of STAT2 protein. Studies by Kumar and co-workers with an Asian lineage virus HPF, Grant and co-workers with an African lineage virus MR766 and Hertzog and colleagues with an Asian lineage fetal brain-isolate, have shown that NS5 mediates the proteasomal degradation of STAT2 in human cell lines^42–44^. In productive ZIKV infection, STAT2 degradation has been shown to occur in Vero cells^43^ and in human lung epithelial A549 and fetal astrocyte HFA cells^42^. In contrast to these observations, Bowen and colleagues reported a lack of STAT2 degradation in live infection in human A549 cells or in human DCs. In fact, the authors observed an increase in STAT2 levels together with other IFN-stimulated genes during ZIKV infection^17^. However, they reported a significant inhibition of STAT1 and STAT2 phosphorylation, which was also similarly observed in the case of STAT1 in the study by Hertzog and colleagues^17,44^. In our study, we saw a clear increase in STAT2 levels together with IFN-induced MxA expression in DCs, which was likely due to ZIKV induced type I and type III IFNs at late time points of infection. The phosphorylated form of STAT2 appeared at 24 h and peaked at 48 h p.i. and then gradually reduced during a 96 h follow-up (Fig. 5A). Before the reduction in STAT2 activation, the expression of viral proteins was observed (Fig. 5A), suggesting that in primary human DCs ZIKV infection is leading to down-regulation of STAT signaling. The role of STAT2 in the pathogenesis of ZIKV has now been proven also with STAT2 knock-out mouse and hamster models^45,46^.

Surprisingly, we observed IRF3 and STAT2 degradation in macrophages at early times of ZIKV infection. This degradation was evident for both African and Asian strains. Macrophages are professional phagocytic cells, and viral recognition leads to an antiviral response including the activation of phagocytosis of the virus and phagolysosome activation. ZIKV NS5 has been associated with proteasomal degradation of STAT2, but this takes place only after viral NS5 protein is expressed^42–44^. It was of interest that the degradation of IRF3 and STAT2 in macrophages took place at early hours of ZIKV infection, clearly before viral protein expression occurred (Fig. 5B). This suggests, that during the ZIKV entry process, the virus is likely activating the host proteasome/lysosome pathways. This finding could provide insights into a completely new evasion strategy of ZIKV where the early events of ZIKV entry trigger the degradation of innate signaling molecules. Other flaviviruses have been shown to utilize various immune evasion strategies in addition of inhibiting the signal transduction by non-structural proteins. For instance, Dengue and Tick-borne encephalitis viruses have been shown to use specific membrane vesicle structures to hide viral replication products in order to evade the host recognition^47,48^. Further studies are warranted to investigate the mechanisms of degradation of host signaling molecules during the early phases of ZIKV infection in human macrophages.

An early degradation of IRF3 and other molecules regulating pathogen recognition could lead to impaired IFN responses during ZIKV infection. In addition of blocking IFN signaling via NS5-mediated degradation of STAT2, several studies have reported an inhibition of RLR signaling suggesting another evasion strategy for ZIKV. With promoter-reporter assays some ZIKV proteins, especially that of NS5 have been associated with an inhibition of IFN gene expression^42,44^. NS1 and NS4B have also been reported to inhibit RLR-mediated IFN gene expression by inhibiting the phosphorylation of TBK1 and IRF3^49^. Thus, the exact molecular mechanisms by which ZIKV is interfering with IFN induction and IFN signaling still needs further analysis in order to understand the pathogenicity of ZIKV and identifying the targets for therapeutic interventions in severe ZIKV infections.

In summary, we show here that recent epidemic Zika virus strains replicate very well in human DCs with strong IFN induction, but their replication is attenuated in macrophages likely leading to impaired host innate immune responses. The infection rate of a historical African ZIKV strain, instead, remains low in both human DCs and macrophages, and the virus activates relatively weak IFN-induced antiviral responses in these cells. In conclusion, our findings indicates that ZIKV evolution has led to cell-type specific differences in the infectivity leading to differential regulation of host innate immune responses in humans.

## Materials and Methods

### Ethics statement

This study was carried out with cultured leukocytes using primary human cells from anonymous buffy coat samples obtained from voluntary adult blood donors from the Finnish Red Cross Blood Service (Blood Service Center Helsinki, Finland). The use of buffy coats for research purposes was approved by the Finnish Red Cross Blood Service Institutional review board (licence number 37/2016, renewed once a year) by which the need for informed consent was waived. Ethical approval for animal immunization was provided by the Ethics committee of animal experimentation in Southern Finland (permission no: ESLH-ESAVI/11411/04.10.07/2014 to DVM Anna Meller). All experimental protocols were approved and performed in accordance with the guidelines of the Finnish Institute for Health and Welfare (THL), Helsinki, Finland.

### Cell cultures

Monocyte-derived dendritic cells (DCs) and macrophages were differentiated from peripheral blood monocytes isolated from the buffy coat fractions collected from healthy blood donors (Finnish Red Cross Blood Service, Helsinki, Finland) according to a standard procedure^50,51^. In brief, peripheral blood mononuclear cells (PBMC) were fractionated by Ficoll-Paque (Pharmacia Biotech) gradient centrifugation. For macrophage cultures, monocytes from the PBMC fraction were allowed to adhere onto cell culture plates and macrophages were differentiated by culturing the cells for 7 days in macrophage serum-free substitution medium (Gibco Invitrogen) supplemented with 0.6 µg/ml penicillin and 60 µg/ml streptomycin in the presence of 10 ng/ml human granulocyte-macrophage colony-stimulating factor (GM-CSF) (Gibco Invitrogen). For DCs, PBMC fraction was subjected to an additional centrifugation on a Percoll gradient (Amersham Biosciences) and lymphocyte depletion with anti-CD3 and anti-CD19 magnetic beads (Dynal). DCs were differentiated by culturing monocytes in RPMI medium (Sigma Aldrich) supplemented with 0.6 µg/ml penicillin, 60 µg/ml streptomycin, 2 mM L-glutamine and 20 mM HEPES in the presence of 10% fetal calf serum (FCS) (Sigma Aldrich), 10 ng/ml GM-CSF (Gibco Invitrogen) and 20 ng/ml IL-4 (GenScript) for 1 week. In all experiments cells from 4 different blood donors were used separately for virus infection and all experiments were repeated several times as indicated in figure legends.

Vero E6 green monkey kidney (ATCC CRL-1586) cells were maintained in Eagle’s minimum essential medium (E-MEM) supplemented with 0.6 µg/ml penicillin, 60 µg/ml streptomycin, 2 mM L-glutamine, 20 mM HEPES and 10% FCS (Sigma Aldrich). *Spodoptera frugiperda* (*Sf*9) cells were used for baculovirus expression and maintained in TNM-FH medium or in EX-C420 Serum-Free Medium (Sigma Aldrich) supplemented with 10% FCS (Sigma-Aldrich) as described previously^52^. Mouse embryonic fibroblast (MEFs) from wild-type mouse and from *Ifnar-1*−/− mouse, both expressing *Mx1*, were kindly provided by Prof. P. Staeheli, Freiburg, Germany. The primary MEFs were immortalized by using a rigorous passaging protocol to obtain wt and KO cell lines. The MEF cell lines were cultured in Dulbecco’s modified Eagle’s Medium (D-MEM) supplemented with 0.6 µg/ml penicillin, 60 µg/ml streptomycin, 2mM L-glutamine, 20 mM HEPES and 10% FCS.

### Viruses and infections

Zika virus strain ZIKV-FB-GWUH-2016 (GenBank number KU870645) was isolated from a fetal brain tissue by Vapalahti and colleagues^5^, and it is a member of an Asian ZIKV genotype. Another recent Asian lineage Zika virus strain ZIKV-H-PF-2013 (GenBank number KJ776791.2), originally a clinical isolate from a viremic adult patient from French Polynesia in 2013, was obtained from European Virus Archive (EVA, Marseille, France). The low-passage-number strain ZIKV-#976-Uganda-1947 is a historical isolate from a monkey and it originates from the European Virus Archive goes global (EVAg, Ref: 007V-EVAg1585). ZIKV strains #976 was isolated in baby mouse brain in 1947 in Uganda and passaged 3 times, then in 1961 it was lyophilised and stored at −80 °C. Then, in 2017 the virus was rehydrated and inoculated on Vero E6 cells. It was propagated in Vero E6 cells 3 times before preparing virus stocks. All Zika virus strains were obtained as Vero E6 cultures and further propagated once for this study resulting in passages p2, p7 or p4 giving TCID_50_ titers of the virus stocks 1.3 × 10^7^ (first GWUH stock) or 1 × 10^8^ (the second GWUH stock), 5.0 × 10^6^ and 2.4 × 10^8^, respectively. A human influenza A virus strain A/Beijing/353/1989 (H3N2) was propagated in 11-days-old embryonated chicken eggs at +36 °C for 3 days and the virus titer in human DCs was 2 × 10^9^ ^21^.

The differentiated DCs and macrophages and immortalized MEFs were infected with indicated ZIKV strains at different MOI values (based on TCID_50_ Vero E6 cell titers), as indicated in the figures. After 2 h of infection at +37 °C fresh media was added and the cells were incubated for different times depending on each experiment. Recombinant human IFN-β (Schering-Plough) was used at 10 or 100 IU/ml cencentrations to pretreat DCs or macrophages for 16 h before ZIKV infection. Infective Zika viruses were handled strictly under Biosafety Level (BSL) 3 laboratory conditions at the Finnish Institute for Health and Welfare (THL), Finland.

### End-point dilution assay

For determining the viral titers from ZIKV samples Vero E6 cells were cultured in 96-well plates. A dilution series of up to 10^−6^ was made from each sample, and each dilution was used to infect 8 parallel culture wells. The cytopathic effect was observed under the light microscope at day 7 post-infection, and each well was scored either positive or negative for virus infection. The Spearman-Karber method was used to calculate the results which are presented as log TCID_50_/ml and the detection limit for the assay was 0.499.

### Antibodies against ZIKV NS3 or NS5 protein

Synthetic ZIKV *NS3* or *NS5* genes were ordered from GenArt (Thermo Fisher Scientific), based on ZIKV GWUH sequence. The coding sequence was cloned into a baculoviral expression vector GST-pBVboost^53^ to produce GST-NS3 or GST-NS5 expressing baculoviruses. For producing recombinant ZIKV proteins *Sf*9 cells were infected with GST-NS3 or GST-NS5 expressing baculoviruses for 42 h. Cells were collected, and ZIKV GST-NS3 or GST-NS5 were purified by glutathione Sepharose affinity chromatography and preparative SDS-PAGE as previously described^53^. The rabbit or guinea pig polyclonal antibodies against ZIKV NS3 or NS5 antigens were prepared by immunizing the animals with purified antigens (50 μg/immunization) for 4 times at 2 week intervals together with Freund’s incomplete (FCA) adjuvant (Difco laboratories). The animals were bled 10 days after the last immunization.

### Immunofluorescence assay (IFA)

For immunofluorescence microscopy cells were grown on glass coverslips for 24 h and infected with ZIKV as indicated in the figure legends. After infection, the cells were fixed with 4% paraformaldehyde at RT for 30 min and permeabilized with 0.1% Triton X-100 for 5 min before staining with anti-ZIKV antisera. Polyclonal rabbit or guinea pig immune sera was used at a dilution of 1:200 for staining the coverslips at RT for 1 h. FITC-labeled goat anti-rabbit or goat anti-guinea pig antibodies (Invitrogen) were used in secondary staining. NucBlue Fixed Cell Ready Probes reagent (Life Technologies, DAPI) was added to secondary antibody staining solutions and slides were analyzed with fluorescence microscopy.

### Flow Cytometry

For infectivity analyses ZIKV-infected DCs were collected from each blood donor separately, cells were fixed with 4% paraformaldehyde at RT for 30 min and permeabilized with 0.1% Triton X-100 for 5 min before staining with anti-ZIKV rabbit antisera and FITC-conjugated secondary antibodies (Invitrogen) at RT for 1 h. Flow cytometry was performed on FACSCanto II cytometer and analyzed using FACS Diva software (BD Biosciences).

### RNA isolation and qRT-PCR

Virus-infected cells were harvested and total cellular RNA was isolated using the RNeasy Mini kit (Qiagen) including DNase digestion (RNase-free DNase kit, Qiagen). One µg of total cellular RNA was transcribed to cDNA using TaqMan Reverse Transcriptase kit (Applied Biosystems) with random hexamers as primers. cDNAs were amplified by PCR using TaqMan Universal PCR Mastermix and Gene Expression Assays (Applied Biosystems). The ZIKV NS5 specific qRT-PCR primers used were modified from Faye *et al*.^54^ and were as previously described^5^. Cytokine or MxA mRNA levels were normalized against human 18S rRNA or mouse Gapdh with TaqMan Endogenous Control kits (Applied Biosystems). Gene expression data is presented as a relative gene expression in relation to unstimulated samples in order to calculate the fold changes seen in infection experiments.

### Immunoblotting

For protein analyses the cells from different blood donors were pooled to get sufficient amounts of proteins, and whole cell lysates were prepared in the passive lysis buffer of Dual Luciferase Assay Kit (Promega) containing 10 mM Na_3_PO_4_. Equal amounts of proteins (10–30 ug/lane) were separated on SDS-PAGE and transferred to Hybond-P polyvinylidene difluoride (PVDF) membranes (Amersham Biosciences). The membranes were blocked with 5% milk protein in PBS. Antibodies against IRF3 and MxA were as previously described^21,22^ and antibodies against ZIKV NS5 were prepared as described above. The staining was done in blocking buffer at RT for 1 h. Antibodies against phosphorylated IRF3 (P-IRF3, #4947), STAT2 (#72604), P-STAT2 (#88410) and GAPDH (#2118) were from Cell Signaling Technology, for β-Actin (SC-10731) from SantaCruz Biotechnology, and the stainings were done in Tris-buffered saline, pH 7.4 containing 5% BSA at +4 °C overnight. HRP-conjugated antibodies (Dako) were used in the secondary staining at RT for 1 h. Protein bands were visualized on HyperMax films using an ECL plus system (GE Healthcare).

### Enzyme immunoassay (EIA)

The secreted levels of IFN-λ1 or multiple subtypes of IFN-α were analysed from cell culture supernatant samples of different blood donors separately using LegendMax^TM^ Human IFN-λ1 ELISA kit (BioLegend) or VeriKine^TM^ Human IFN-α Multi subtype ELISA kit (PBL Assay Science), respectively.

## Supplementary information

Supplementary Figure S1.

## Data Availability

All the research material and the data produced in this study are available from the corresponding author upon request following the practices of our institute.
