# Supplementary material for: Asian and African lineage Zika viruses show differential replication and innate immune responses in human dendritic cells and macrophages
Source: Sci Rep. 2019 Oct 31;9:15710. doi: 10.1038/s41598-019-52307-1 (PMC6823455; doi:10.1038/s41598-019-52307-1)
Supplement: Supplementary file 1 — Supplementary Figure S1. [file 41598_2019_52307_MOESM1_ESM.pdf]

Asian and African lineage Zika viruses show differential replication and innate immune responses in human dendritic cells and macrophages

Pamela Österlund, Miao Jiang, Veera Westenius, Suvi Kuivanen, Riia Järvi, Laura Kakkola, Rickard Lundberg, Krister Melén, Miša Korva, Tatjana Avšič – Županc, Olli Vapalahti, and Ilkka Julkunen

Supplementary Figure S1

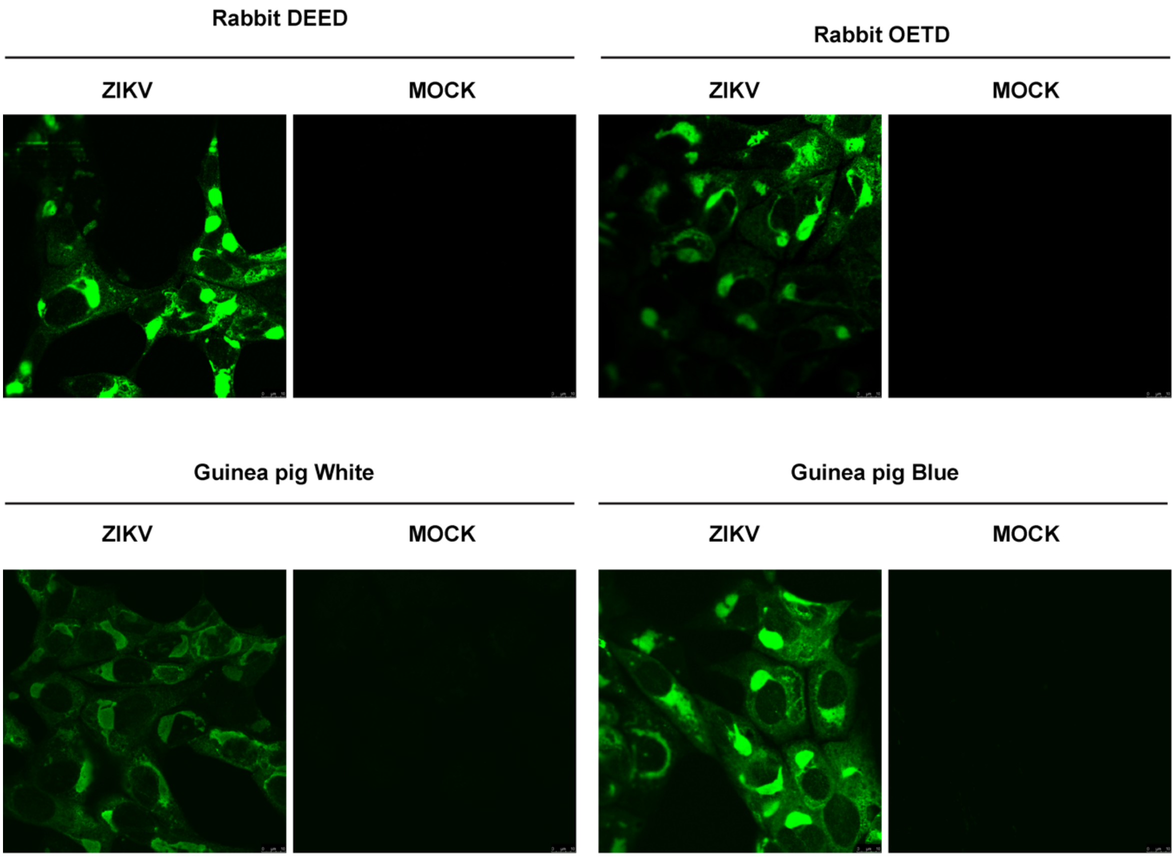

Supplementary Figure S1. Specificity of rabbit and guinea pig polyclonal antibodies against ZIKV NS3 protein in ZIKV-infected cells. VeroE6 cells grown on glass cover slips were either mock infected or infected with a ZIKV GWUH strain at MOI of 2 for 4 days. The cells were fixed, permeabilized and stained with NS3-specific immune sera from two different rabbits or two guinea pigs at dilutions of 1:200. The immunofluorescence straining patterns of NS3 was analyzed by confocal laser microscopy.
